# Giant Left Ventricle Outflow Tract Pseudoaneurysm after Ross Procedure

**DOI:** 10.5935/abc.20170008

**Published:** 2017-04

**Authors:** Sílvia Leão, Sofia Carvalho, Hélder Ribeiro, Paulo Fontes, J. Ilídio Moreira

**Affiliations:** Centro Hospitalar de Trás-Os-Montes e Alto Douro, Hospital de Vila Real, Portugal

**Keywords:** Ventricular Outflow Obstruction / surgery, Cardiac Surgical Procedures / complications, Echocardiography

A 33-year-old woman was admitted to our hospital because of dyspnea on exertion, orthopnea, cough and pedal edema for the past six months. Six years earlier she had been submitted to Ross procedure for correction of a bicuspid aortic valve.

Physical exam was unremarkable except by a grade 3 systolic murmur on left sternal border.

**Figure 1:**
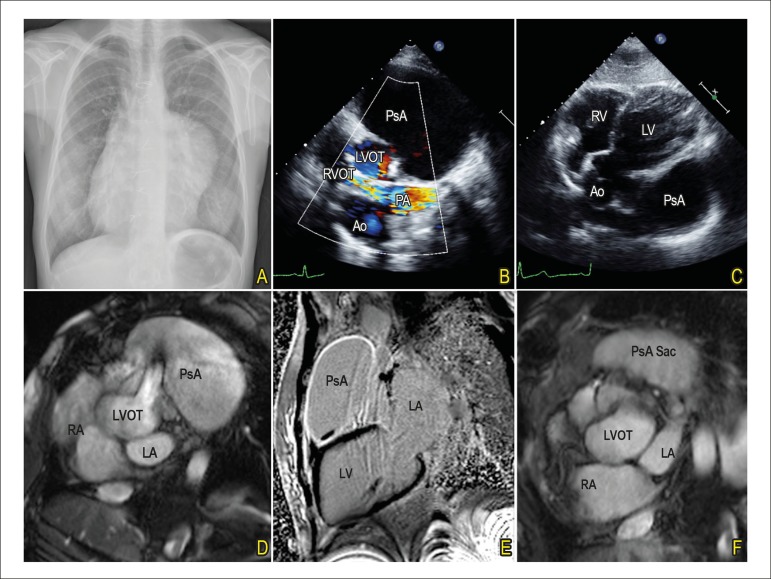
A) Chest radiograph; B and C) Preoperative transthoracic echocardiography, modified short axis and subcostal views; D and E) Preoperative magnetic resonance imaging; F) Follow-up magnetic resonance imaging. Ao: aorta; LA: left atrium; LVOT: left ventricle outflow tract; LV: left ventricule; RA: right atrium; RV: right ventricule; PA: pulmonary artery; PsA: Pseudoaneurysm; RVOT: right ventricle outflow tract.

A chest X-ray revealed an opacity on left border of cardiac silhouette (Figure 1A). Transthoracic echocardiography presented a giant saccular structure, adjacent and connected to the left ventricular outflow tract through a neck located at 2 o´clock position, compatible with a pseudoaneurysm. This structure caused compression of the right ventricle outflow tract (RVOT) and pulmonary artery, causing mild obstruction (Figure 1 B and C).

Cardiac magnetic resonance imaging identified its origin on left ventricule outflow tract (LVOT), in close relation to left and non-coronary sinus. There were small thrombi inside the pseudoaneurysm, tapering the wall adjacent to the left ventricle (Figures 1D and E).

Patient was surgically treated. The pseudoaneurysm sack was opened through a transpleural access and the communication between the LVOT and the pseudoaneurysmal cavity was closed with a Teflon patch. Patient's post-operative recovery and follow-up were uneventful.

After 3 months of follow-up she is asymptomatic. The pseudoaneurysm is completely excluded from arterial circulation, without significant obstruction of RVOT (Figure 1F).

LVOT pseudoaneurysm is an uncommon but potentially life-threatening complication of Ross procedure. Follow-up with imaging techniques allows early identification and prompt intervention.

